# Application research of computational mass-transfer differential equation in MBR concentration field simulation

**DOI:** 10.1186/s40064-016-2324-0

**Published:** 2016-06-16

**Authors:** Chunqing Li, Xiaobo Tie, Kai Liang, Chanjuan Ji

**Affiliations:** School of Computer Science and Software Technology, Tianjin Polytechnic University, Tianjin, China

**Keywords:** MBR, Mass-transfer differential equation, Velocity field, Concentration field

## Abstract

After conducting the intensive research on the distribution of fluid’s velocity and biochemical reactions in the membrane bioreactor (MBR), this paper introduces the use of the mass-transfer differential equation to simulate the distribution of the chemical oxygen demand (COD) concentration in MBR membrane pool. The solutions are as follows: first, use computational fluid dynamics to establish a flow control equation model of the fluid in MBR membrane pool; second, calculate this model by adopting direct numerical simulation to get the velocity field of the fluid in membrane pool; third, combine the data of velocity field to establish mass-transfer differential equation model for the concentration field in MBR membrane pool, and use Seidel iteration method to solve the equation model; last but not least, substitute the real factory data into the velocity and concentration field model to calculate simulation results, and use visualization software Tecplot to display the results. Finally by analyzing the nephogram of COD concentration distribution, it can be found that the simulation result conforms the distribution rule of the COD’s concentration in real membrane pool, and the mass-transfer phenomenon can be affected by the velocity field of the fluid in membrane pool. The simulation results of this paper have certain reference value for the design optimization of the real MBR system.

## Background

Membrane bioreactor (MBR), a new water treatment technology which was developed based on the traditional biological treatment process that combined with membrane separation technology. Based on the interception of the membrane separation effect, MBR has basically solved a variety of problems of the traditional activated sludge process, and it has enormous performance advantages that the conventional activated sludge process does not have. As can be seen from the phenomenon of membrane separation, under the impetus of the external force, all of the small molecular components of the material liquid can go through the separating membrane component freely. However, as a result of the screening and filtration effect, it makes some of the larger colloidal particles accumulate and concentrate in the region approach the membrane surface which thereby forms a concentration gradient between the material liquid on the film surface and the mainstream body material liquid; and then forms a stable concentration polarization boundary layer in the region close to the membrane surface which is the so-called concentration polarization. Therefore, the accurate simulation for the concentration of the membrane pool is a vital link to reduce the phenomenon of concentration polarization because they can prevent or reduce the problem of membrane fouling in MBR to a certain degree.

Computational mass transfer (CMT) is a set of theories and methods that references computational heat transfer (CHT) and computational fluid dynamics (CFD) strictly considers temperature distribution, velocity distribution, biochemical reaction, and finally makes the quantitative description of concentration distribution on mass transfer state (Liu et al. 2004). Some researchers have used CMT to simulate concentration field, and although the calculation results are of corresponding deviation for different models, they are mostly consistent with the theoretical values. It proves that the CMT can be applied to the simulation of concentration field very well (Liu et al. 2008).

There are no relevant literatures about the application of CMT on MBR field. However, in the field of chemical industry, especially about the rectifying tower, there are a lot of articles about the application of CMT on the concentration field simulation. Both MBR and distillation tower follow the momentum transfer rule and concentration transfer rule, therefore, during the research of MBR, the CMT models on chemical industry can be consulted. After obtaining the velocity field in MBR membrane pool, CMT is used to simulate the concentration distribution in membrane pool, and Tecplot is used to display the simulation result to get a clear visualization of diagrams about the macro concentration change in MBR membrane pool.

## The simulation of MBR velocity field

### Mathematical model of MBR velocity field

The control equation of CFD mainly includes the mass conservation equation (also known as the continuity equation), momentum conservation equation, and energy conservation equation. Because the fluid in MBR mainly consists of liquid and solid, and the concentration is almost constant, the problem of fluid energy conversion needs not to be considered. The mass conservation equation and momentum conservation equation are used during the process of practical application, hence the equation that Xinliang Li had published in 2001 in the Acta Mechanica Sinica journal is used in this article. The equation is used to simulate the two-dimensional channel flow, and the specific form are as follows:

1 $$\frac{\partial u}{\partial x} + \frac{\partial v}{\partial y} + \frac{\partial w}{\partial z} = 0$$

2 $$\begin{aligned} \frac{\partial u}{\partial t} = - \frac{\partial P}{\partial x} + \frac{1}{\text{Re}}\frac{\partial u}{\partial x} + udiv(U) + f\overrightarrow {{e_{x} }} \hfill \\ \frac{\partial v}{\partial t} = - \frac{\partial P}{\partial y} + \frac{1}{\text{Re}}\frac{\partial v}{\partial y} + vdiv(U) + f\overrightarrow {{e_{y} }} \hfill \\ \frac{\partial w}{\partial t} = - \frac{\partial P}{\partial z} + \frac{1}{\text{Re}}\frac{\partial w}{\partial z} + wdiv(U) + f\overrightarrow {{e_{z} }} \hfill \\ \end{aligned}$$

Among the equation, $f$ is the mean pressure gradient, $\overrightarrow {e}$ is the unit vector corresponding to coordinate axes, $div(U) = \frac{\partial u}{\partial x} + \frac{\partial v}{\partial y} + \frac{\partial w}{\partial z}$. Dimensionless parameter is defined as: $\text{Re} = \frac{{U_{m} \delta }}{v}$, $U_{m} = \frac{Q}{2\delta }$ is average flow rate, $Q$ is the amount of flow in channel, $\delta$ is the half-width of channel, $v$ is fluid viscosity.

The method of efficient direct numerical simulation (DNS) is adopted in this paper to solve the equations. It was published in the Journal of Chinese Journal of Theoretical and Applied Mechanics in 2001 by Li (2001). It uses discrete pressure Pioson equation which is based on staggered grids to calculate the pressure term, and it solves the problem of residual divergence around the simulation border; at the same time, it uses fast Fourier transform to calculate part of the implicit equation; in the end, it uses chasing method to calculate the discrete algebraic equations which greatly reduces the computation amount. By using this method to get the numerical simulation for the fluid velocity field in two-dimensional channel, simulation results show that this method is of good stability, high precision, and it can also suppress aliasing errors. In addition, this method is of high computation efficiency and is effective to simulate the turbulent wall flow.

### Grid division

According to the established mathematical model and the solution method used in part 1.1, the non-equidistant mesh division in two-dimensional model is adopted during the process of calculation in this chapter. Between the up and down plates of the two-dimensional channel flow, there is full of flowable and incompressible viscous fluid. Under the uniform pressure gradient, the fluid flows along the X direction.

**Fig. 1:**
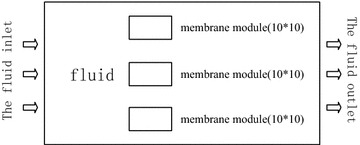
Schematic diagram of MBR simulation area

Considering that the flow process of MBR is equivalent to all the liquid flows along the membrane modules around, the liquid penetrates into the hollow fiber membrane in the membrane modules. During the process of simulation, in order to better apply the algorithm and accurately simulate the process of the flow of the membrane pool, three membrane modules are placed at the middle of simulation area in the channel (placed side by side along y-axis). The membrane modules selected are 10 × 10 hollow fiber membrane. The design of the simulation area is roughly shown as Fig. 1.

**Fig. 2:**
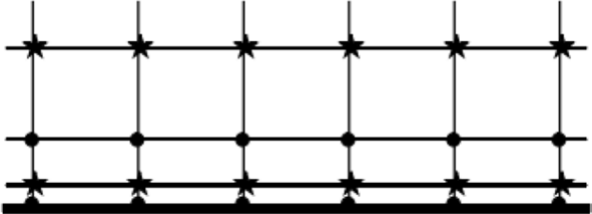
Grid division figure

The grid division method of this paper is shown in Fig. 2: in the flow direction, grids are divided uniformly while in the normal direction divided non-uniformly. Black dots in Fig. 2 represent the grid points of velocity v, and solid five-pointed stars represent the grid points of velocity u and p. Because the grid points in the x direction are uniform, the position of the grids can be calculated successively by dividing the total length by the number of grids. While the grid points in the y direction are non-uniform, the position of them can be calculated by the following formula:

- the grid point of v:
  $$y_{j} = - 1 + \tanh \left( {b_{g} (j - 1)\frac{2}{N - 1}} \right)/\tanh (b_{g} )\quad (j = 1,2,3, \ldots ,N)$$
- the grid point of u, p:
  $$y_{j} = - 1 + \tanh \left(b_{g} \left(j^{'} - \frac{1}{2}\right)\frac{2}{N - 1}\right)/\tanh (b_{g})\quad (j^{'} = 1,2,3, \ldots ,N - 1)$$

where the dimensionless length selects 2.

### Boundary conditions

When solving the flow direction in simulation area, the periodic boundary conditions are adopted. As a result, during the calculation process, the velocity of left and right entrance is affected by the value of each other, that is:

3 $$\begin{aligned} U_{0,j} & = U_{nx,j} \\ U_{nx + 1,j} & = U_{1,j} \\ \end{aligned}$$

where U is the velocity vector, grid points in x and y direction both start counting from 1. $U_{0,j}$ and $U_{nx + 1,j}$ represent the boundary values of left boundary and right boundary.

For the points on the upper and lower wall surface of membrane pool and the points in the membrane modules, no-slip boundary condition is set, which means that the velocity in each direction is 0:

$$\begin{aligned} U_{i,1} & = 0 \\ U_{i,ny} & = 0 \\ \end{aligned}$$

For the boundary points around membrane modules, because of existence of the penetration, the quality of the boundary points decline when they flow into the modules. Therefore, for these boundary points, it is necessary to add a certain negative value to the right of mass conservation equations to indicate penetration. The negative value can be calculated according to the amount of the flow of membrane modules. Then the mass conservation equation is modified as following:

4 $$\frac{\partial u}{\partial x} + \frac{\partial v}{\partial y} + \frac{\partial w}{\partial z} = d$$

## The simulation of MBR concentration field

### Mathematical model of MBR concentration field

There were no relevant literatures published about the application of computational mass transfer theory in the field of MBR, so there is not any literature reference for the establishment of mathematical model in this field. However, in the field of chemical industry, especially the rectifying tower, there are a lot of literatures about concentration field simulation. Therefore, in the field of MBR, mathematical model of the chemical industry can be used. Because both the rectifying tower and the MBR follow the law of momentum transference and concentration transference, the model of computational mass transfer theory is commonly accepted.

Yu et al. (1990) combined mass transfer equation and momentum transfer equation to simulate two-dimensional velocity field, and then he used mass transfer equation to obtain the concentration distribution of trays. Mass transfer equation is:

5 $$u_{x} \frac{{\partial X_{A} }}{\partial x} + u_{y} \frac{{\partial X_{A} }}{\partial y} = D_{e} \frac{{\partial X^{2}_{A} }}{{\partial x^{2} }} + D_{e} \frac{{\partial X^{2}_{A} }}{{\partial y^{2} }} + \frac{{k_{g} a}}{m\rho }X_{A}$$

$$X = \frac{{x_{A} - x^{*} }}{{x_{m} - x^{*} }}$$

where diffusion coefficient $D_{e}$ was approximately set as $D_{e} = 1.25v_{e}$.

Botan Liu used fluid dynamics software PHEONICS to calculate the three-dimensional concentration field of trays (Botan 2003). Mass transfer equation is:

6 $$u_{x} \frac{{\partial X_{A} }}{\partial x} + u_{y} \frac{{\partial X_{A} }}{\partial y} + u_{z} \frac{{\partial X_{A} }}{\partial z} = D_{e} \frac{{\partial X^{2}_{A} }}{{\partial x^{2} }} + D_{e} \frac{{\partial X^{2}_{A} }}{{\partial y^{2} }} + D_{e} \frac{{\partial X^{2}_{A} }}{{\partial z^{2} }} + S_{c}$$

where *X*_*A*_ is the liquid concentration of light components. Assuming the gas is completely mixed, the mass transfer source term $S_{c}$ is:

7 $$S_{c} = \frac{{\rho_{G} }}{{\rho_{L} }}k_{L} a(x^{*} - x_{A} )$$

Using this model, the calculation result is basically consistent with the prediction result.

This paper only considers the single-phase state in MBR membrane pool, so only the diffusion process of molecular needs to considered. For the biochemical reactions (the reactions of microbial decomposing COD) in the membrane pool, source term is used to indicate in this paper. Three-dimensional mass transfer equation is:

8 $$u_{x} \frac{\partial C}{\partial x} + u_{y} \frac{\partial C}{\partial y} = D_{e} \left(\frac{{\partial^{2} C}}{{\partial x^{2} }} + \frac{{\partial^{2} C}}{{\partial y^{2} }}\right) + S_{c}$$

where C is the concentration of fluid components. For the biochemical reactions, linear manner is used to indicate:

9 $$S_{c} = \frac{\partial C}{\partial t} = AC$$

where the value of A can be calculated according to the residence time of the liquid.

### Solve the mathematical model of MBR concentration field

When solving the mass transfer equation of concentration field, the first-order partial differential uses front differential to decompose while the second-order partial differential uses center differential to decompose. After decomposition, the number of equations is the product of grid number in x direction and y direction. The unknown parameters in these equations are the concentration of each grid point, so the number of unknown parameters is same as the number of equations, which implies that the concentration of each grid point can be calculated by solving these equations. The coefficients on the right of these equations are all zero, so the equations are homogeneous and can be solved by LU decomposition and iterative method.

LU decomposition method was considered to solve problems, however, there are a lot of grid decomposition on the macro dimensions, which makes the array size exceed the computer’s memory. For example, if the number of matrix of 128 × 129 is used, the concentration of each point will have a total of 16,512 unknowns. According to the solve of mass transfer equation, there will be 16,512 equations which makes the number of LU matrix reach 272,646,144. If floating-point type storage is used, each numeral occupies four bytes in length, then 260 G memory is needed. This is only a conservative estimate, and it is very likely other unknown overhead may show up during the process of calculating. Therefore, this method is not applicable to this article.

Taking the memory into account, iterative method that occupies less memory is selected in this paper. However, compared with LU decomposition, it takes more time and converges slower; moreover, it is not as good as LU decomposition on obtaining the exact solution directly. To improve convergence speed, Seidel iterative method is used in this paper. During the calculation process, it can be found that for the length of the grid above, it can be accurate to 10 × *E*−7 till the iteration times is about $10^{5}$; moreover, when the grid number is less, calculation result of this method is almost equal with LU decomposition, whose deviation is less than 10 × *E*−4. So Seidel iteration can be well applied to this article.

### Boundary conditions

In part 1.3, the boundary condition in flow direction is periodic. That is the fluid flowing from the inlet to the outlet cyclically. Upper and lower boundary are treated as wall surface.

When simulating the concentration field, the data in velocity field is needed, so the boundary conditions need to be consistent. The upper and lower boundary are treated as wall surface, so their velocity is set as 0. If not consider the vertical transfer process on the wall surface, but only consider the horizontal, then the concentration on the wall surface is not calculated by the concentration in normal direction but by the concentration in flow direction and the biochemical reactions occurred here. Calculation equation is:

10 $$u_{x} \frac{\partial C}{\partial x} = D_{e} \frac{{\partial^{2} C}}{{\partial x^{2} }} + S_{c}$$

Assuming the concentration distribution around the inlet and outlet is uniform, then their concentration is set as:

$$\begin{aligned} C = C_{in} \hfill \\ C = C_{out} \hfill \\ \end{aligned}$$

### Set the source term

In part 2.1, the biochemical reaction in membrane pool is proceed with the source term as follows:

$$S_{c} = \frac{dC}{dt} = AC$$

Assuming it takes time *t for* the fluid flows from inlet to outlet. Inlet concentration and outlet concentrations are $C_{in}$, $C_{out}$, respectively, then the above equation can be derived as follows:

- transposition for the formula: $\frac{1}{C}dC = Adt$
- set *t* as upper limit, integrating result: $\mathop \int \nolimits_{0}^{t} \frac{1}{C}dC = \mathop \int \nolimits_{0}^{t} Adt$
- solving the integral: $(\ln C)_{0}^{t} = (At)_{0}^{t}$
- the final result: $C_{out} = C_{in} e^{At}$
- the source term is:
  11 $$S_{c} = \ln \left(\frac{{C_{out} }}{{C_{in} }}\right)/t$$

The concentration of inlet and outlet can be obtained from experiment, and the value of $t$ can be obtained from the residence time of fluid in MBR membrane pool.

## Simulation experiment

In this article, the experimental data was extracted from industrial production and test data of a MBR wastewater treatment in Shijiazhuang. The membrane modules are PVDF hollow fiber microfiltration membrane modules which were made by Tianjin MoTianMo Engineering Co. The fiber is of 2 mm aperture, external pressure water, effective use of area is 40 m^2^.

### MBR velocity field simulation experiment

The key data required in velocity field simulation as shown in Table 1. The specific data required in grid division as shown in Table 2.

**Table 1:** Data needed in velocity field simulation

Membrane modules number	3
Hollow fiber membrane number in each module	7500
Total amount of inflow (outflow)	1 m^3^/h
Outside diameter (diameter) of hollow fiber membrane	1.5 mm

**Table 2:** Part of grid data in velocity field simulation

Simulation area size	2 × 2π (dm × dm)
Grid number	256 × 258
Membrane module size	0.2 × 0.2 (dm × dm)
Position of membrane module	Side by side at the 2 dm
Reynolds number	1400
Step length (dimensionless)	0.004
Total time (dimensionless)	20
Amount of flow in simulation area	4/3

The initial value of flow field:

$$\begin{array}{ll} {u(i,j) = 1 - y^{2} } \\ {v(i,j) = 0} \\ \end{array},$$

where y is a number between 0 and 1. The value of u is 0 at the boundary, and 1 at the middle of entrance, and set according to its y coordinate at other points.

For convenience in calculations, the unit of speed is set as dm/s. However, in reality, the fluid in MBR membrane pool flows slowly and the unit is about cm/s. Therefore, during the simulation in this article, the speed is ten times as the actual value. When displaying in result, the speed is decreased to normal according to corresponding scaled.

During the simulation, in order to simulate the process of penetration around membrane modules, it assumes that the flow amount around membrane modules is reduced, so it is necessary to calculate the value of *d*. Outflow is 1 m^3^/h, and the total number of membranes is 7500 × 3; the membrane modules are placed by 10 × 10, so the permeation amount around each membrane module is about 0.00123 dm^3^/s. The height of membrane pool is 1 m, then the permeation amount of each membrane module is about 1.2 × 10^−4^ dm^3^/s in two-dimensional case. Because the simulation speed is 10 times fast as the actual value, the amount of the permeation is also 10 times large as the actual value. The length and width of membrane module is set to 0.2 dm, the total penetration length is 0.8 dm. After calculation, the unit permeation amount is 0.0015, so the value of *d* in Eq. (4) is set to $- 0.0015$.

**Fig. 3:**
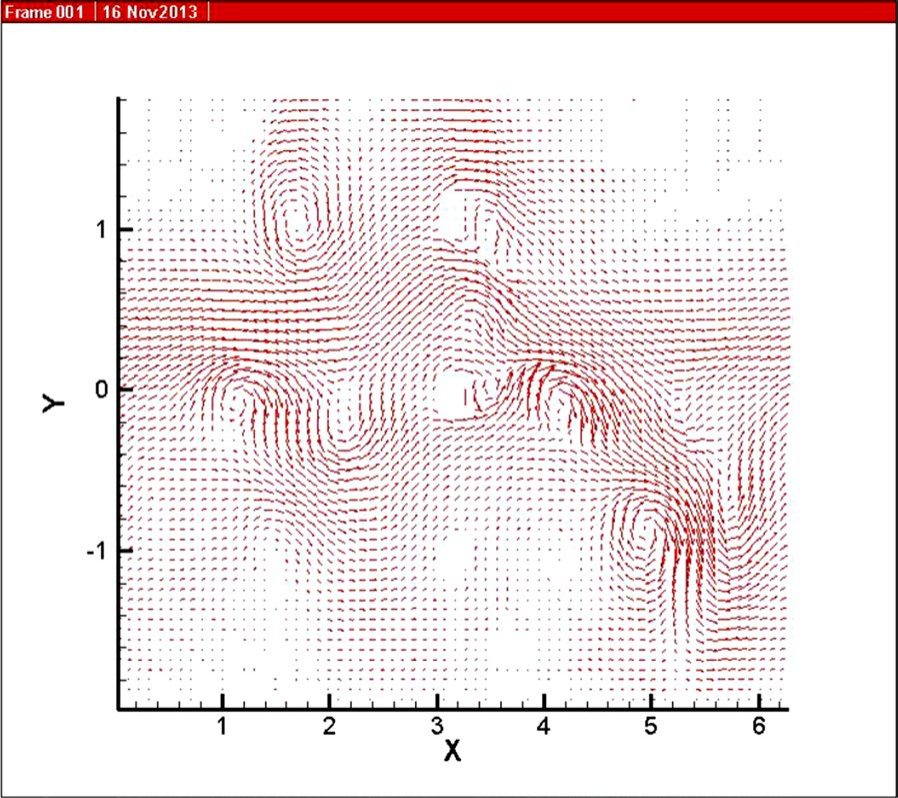
Velocity vector in simulation area

Tecplot is used to visualize the simulation result, Fig. 3 shows the vector illustration of the velocity in the whole simulation area, where the unit is cm/s, the size of simulation area is 4 × 2π. As it can be seen in Fig. 3, the outlet velocity is slower than the inlet in the membrane module. The reason is that the permeation amount around membrane modules is small, so during the flowing process, certain conditions of wall surface have affected the fluid flowing along the flow direction, which makes small area reflux on the left of membrane module as shown in Fig. 3. In addition, the closer to upper and lower boundary the smaller of the speed is. It shows that the simulation result is affected by the condition of wall surface set above. Therefore, the simulation result matches the actual rules of flow.

### MBR concentration field simulation experiment

In this article, concentration field simulation is mainly used to simulate the COD distribution in MBR membrane pool, so only those COD concentrations in membrane pool and at the outlet are selected. For the velocity field data used in CMT, the experimental result in part 3.1 is adopted. Other data needed in concentration field simulation is consistent with part 3.1.

As it can be seen from the mass transfer equations, unknown parameters are diffusion concentration, source term and the concentration of inlet and outlet in membrane pool. Because the impurities amount of water is low in membrane pool, this article selects the common diffusion coefficient 10 × *E*−5 m^2^/s. By analyzing MBR experimental data, it can be found that the COD concentration in membrane pool is about 100, the outlet COD concentration is about 30. Therefore, the inlet COD concentration and the outlet COD concentration are set as 102 and 30 mg/L respectively. The value of source term depends on the concentration of inlet and outlet and the residence time of the fluid. When simulating the velocity field, the width of flow is 2π dm and the actual velocity is 1 cm/s, which gives source term a number of $- 0.00582$ after being calculated.

In both flow and normal directions, grid division here is uniform. The grid number is consistent with the velocity field simulation, which is 256 × 258.

**Fig. 4:**
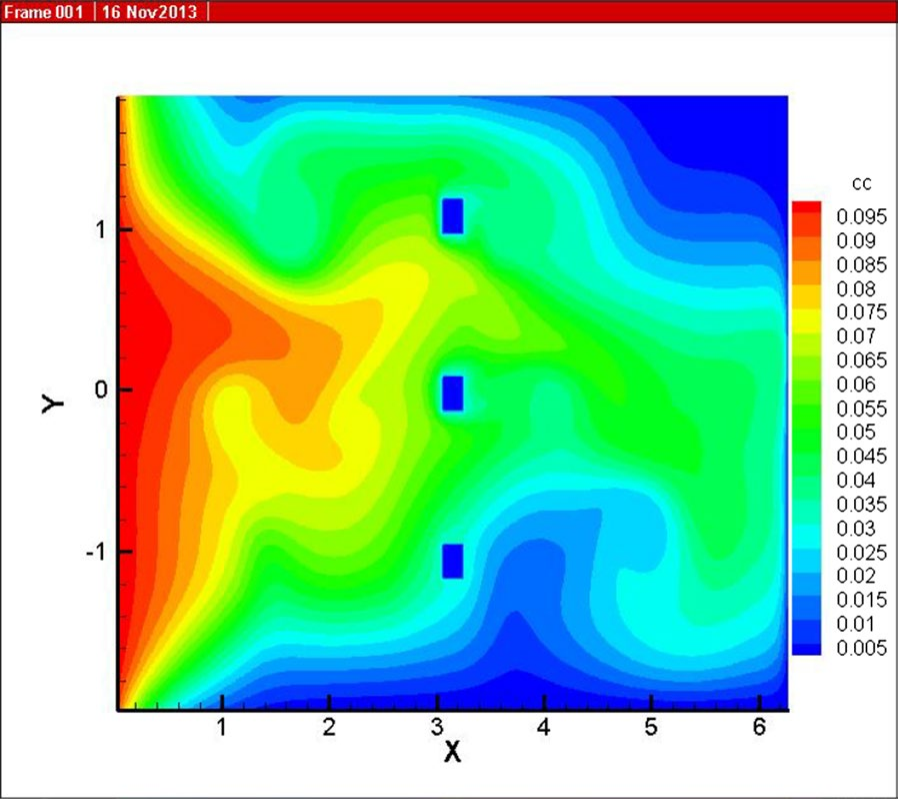
Concentration distribution of COD

By using Tecplot to visualize the simulation result, simulation diagram can be shown as in Fig. 4. In Fig. 4, x-axis represents the flow direction, y-axis represents the normal direction. Upper and lower boundary is wall surface. The position $x = 0$ represents the inlet, and the initial concentration is set at 0.1. When $x = 2\pi$, there is no special treatment. The grids around membrane modules (approximately $x = \pi$) are processed with outlet condition and the concentration is set to 0.03. Simulation error is set to 10 × *E*−7.

By observing the Fig. 4, the following rules can be found: COD concentration in x-axis (the flow direction) shows a decreasing trend, which proves that the COD concentration is affected by biochemical reactions during the flow process, so it proves that the fluid concentration is affected by source term in x-axis. When the x coordinate is kept same, the closer it is to the upper and lower boundary, the smaller is the concentration. Since during the mass transfer process, the concentration is not only affected by diffusion coefficient but also by the fluid velocity, the faster the flow speed is, the slower the change of the fluid concentration is. It can be known from the experiment result in part 3.1, affected by wall surface, the closer it is to the upper and lower boundary, the slower the flow speed is, which shows the obvious reduction of concentration. The experiment results completely match the rules of concentration that is affected by the fluid velocity.

## Conclusion

After studying the change rule of each factor in practical wastewater treatment system as well as basing on the intensive research on the Computational Fluid Dynamics, Computational Mass-Transfer and the MBR, this article takes the process that using MBR to deal with the problem of refractory organic industrial wastewater as research object, and uses mass conservation equation and momentum conservation equation in CFD to build the model of fluid velocity field in MBR membrane pool. Combining the result of velocity field simulation and using three-dimensional mass transfer equations, it built the mathematical model for concentration field in MBR membrane pool, and elaborated the mathematical methods used to solve the model. Then it substituted real factories data into velocity field model and concentration field model and calculated the result. Furthermore, it used the software of Tecplot to visualize the simulation results. By observing the figures of simulation results, it can be found that velocity field simulation result matches the rules of fluid flowing in real membrane pool; COD distribution simulation result reflects that concentration can be affected not only by biochemical reactions but also velocity of flow. By using the research result of this article, the design of real MBR system can be optimized, which can relieve the degree of membrane fouling.
